# Incidence and Prevalence of Multiple Sclerosis in Malmö, Southern Sweden

**DOI:** 10.1155/2022/5464370

**Published:** 2022-03-19

**Authors:** Lucía Alonso-Magdalena, Elisabet Zia, Olga Carmona i Codina, Hélène Pessah-Rasmussen, Peter Sundström

**Affiliations:** ^1^Department of Neurology, Skåne University Hospital and Department of Clinical Sciences, Lund University, Lund, Sweden; ^2^Department of Clinical Sciences, Lund University, Malmö, Sweden; ^3^Department of Neurology, Fundacio Salut Emporda, Figueres and Department of Clinical Sciences, Faculty of Medicine, Girona University, Spain; ^4^Department of Rehabilitation medicine, Skåne University Hospital and Department of Clinical Sciences, Lund University, Lund, Sweden; ^5^Department of Clinical Science, Neurosciences, Umeå University, Umeå, Sweden

## Abstract

**Objectives:**

To estimate the incidence and prevalence of multiple sclerosis (MS) in Malmö municipality in southwestern Sweden.

**Materials and Methods:**

Multiple sources were used in the case identification process. Case ascertainment was assessed by medical chart review including examinations such as magnetic resonance imaging, cerebrospinal fluid analyses, and relevant laboratory tests. Cases were classified according to the 2010 McDonald's diagnostic criteria. Onset-adjusted prevalence and a definition of onset symptoms were applied.

**Results:**

The crude incidence of MS in 2001-2010 in Malmö municipality was 5.3/100,000 (95% confidence interval (CI): 4.5 to 6.2). There was a relapsing onset in 90.5% of cases. The female to male ratio was 1.8. The onset-adjusted prevalence for Dec 2010 was 133/100,000 (95% CI, 120 to 146) with a female to male ratio of 2.1.

**Conclusions:**

This is the first population-based epidemiological study in Skåne, the most southwestern part of Sweden showing a high incidence and prevalence. We found a lower incidence than expected according to previous nationwide figures, probably due to methodological differences between the studies. Our findings support the presence of a north-south gradient of MS prevalence in Sweden.

## 1. Introduction

The most striking epidemiological characteristic of multiple sclerosis (MS) is the uneven distribution of the disease across the world. The disease is particularly prevalent where white people of Nordic origin live, in temperate zones, and in high-income countries. Conversely, MS is uncommon where nonwhites live, in low-income countries, and in tropical zones [1]. The traditional view is that there is a latitudinal gradient with higher latitude correlating with higher prevalence and incidence [2]. This well-accepted theory of a latitudinal gradient has been challenged in the last decades with the gradient being dismissed regarding Europe and North America but still apparent for New Zealand and Australia, and with age and gender adjustments partially eliminating the latitude effect [1, 3]. However, other meta-analyses performed more recently have confirmed both the latitude gradient for incidence in Europe and the association between MS prevalence and latitude globally [4, 5].

The prevalence of MS in Sweden in 2008 has been estimated to be 189/100,000 with a north-south gradient ranging between 227 and 168/100,000 [6]. The average MS incidence in Sweden from 2001 to 2008 has been estimated to be 10.2 per 100,000 [7]. However, both nationwide studies used only register data, and no validation of diagnoses was done. The prevalence of MS in Västerbotten County, northern Sweden, in 2010 has been estimated to be 215/100,000, and the incidence during the period 1998-2010 was 6/100,000; in this population-based study, the diagnosis had been validated [8]. There are other population-based studies of MS incidence and prevalence in Sweden [9–12], but studies from Skåne at the most southwestern part of the country are lacking.

The aim of this study was to determine the incidence in 2001-2010 and the prevalence of MS on 31 December 2010 in Malmö municipality in southwestern Sweden and to provide a base for further follow-up studies.

## 2. Material and Methods

### 2.1. Study Area and Population

**Figure 1:**
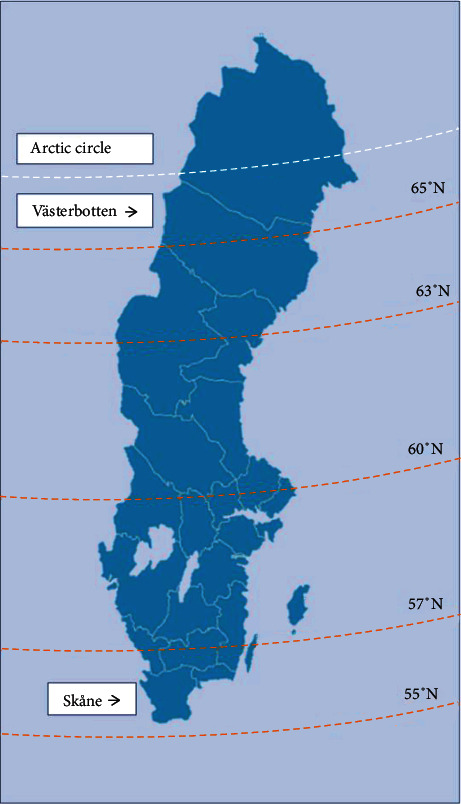
Map of Sweden (adapted from https://Regionfakta.com).

**Figure 2:**
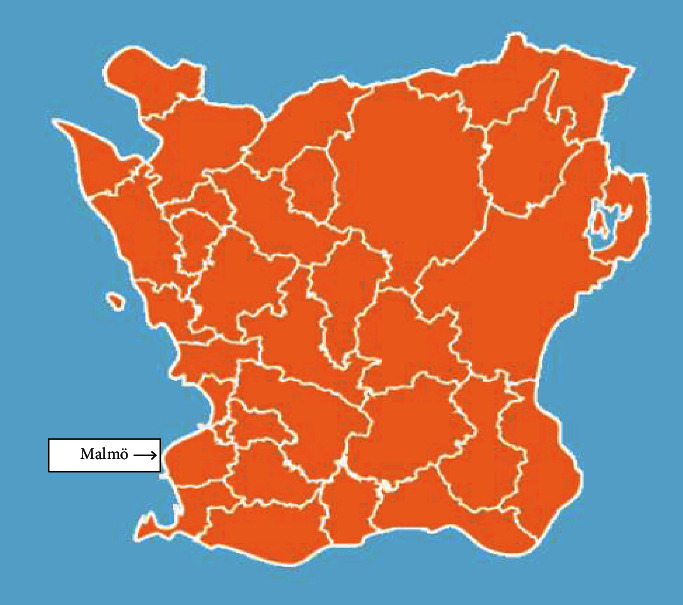
County of Skåne and Malmö municipality (adapted from https://Regionfakta.com).

Malmö municipality, or City of Malmö, is a Swedish municipality located in the county of Skåne in the southwest of Sweden at 55°35′ N latitude having a population of 298,963 persons on the prevalence day, 31 December 2010, in an area of 335 km^2^ (Statistics Sweden; https://www.scb.se) (Figures 1 and 2).

The population of Malmö has been attending the Lund University Hospital Department of Neurology until 1984 when a neurological department was established at the Malmö University Hospital. In 2010, the Skåne University Hospital was established by merging these two hospitals and a rehabilitation hospital (Orup Hospital). Furthermore, there is a local hospital in Trelleborg, 30 km south from Malmö, with neurologist since 1999. Most MS care in Sweden is in the public specialized health service. However, there have been a few private neurologists in Skåne with the one based in Malmö not focused on MS.

### 2.2. Case Definition

The concept of onset-adjusted prevalence was applied [13]. A prevalent case was defined to be an individual that had experienced an onset symptom in agreement with the definition before the prevalence day, was residing in the study area on the prevalence day, and fulfilled the 2010 McDonald's diagnostic criteria for MS at the time for case ascertainment [14]. An incident case was defined to be an individual that had experienced an onset symptom in agreement with the definition when residing in the study area any time from 1 January 2001 to 31 December 2010 and had been later judged to fulfill the 2010 McDonald's diagnostic criteria [14]. Since the McDonald's diagnostic criteria do not define what should be considered as the first manifestation, we adopted a list of symptoms for that purpose [15]. This was used with one minor modification that “myelitis” was used instead of “transverse myelitis.”

The pattern and course of the disease were defined according to Lublin et al. [16].

### 2.3. Case Retrieval

Four sources were used to identify MS cases:

1. The Swedish National Patient Register (NPR) at the National Board of Health and Welfare (http://www.socialstyrelsen.se) contains information on all inpatient care from 1987 and also outpatient visits from 2001. The NPR was searched for patients attending either public (Malmö University Hospital, Lund University Hospital, Orup Hospital, Trelleborg Hospital, and Skåne University Hospital) or private (Malmö, Lund, and Helsingborg) caregivers and diagnosed with MS, demyelinating disorders in CNS, optic neuritis, spastic paraplegia, ataxia, myelopathy, spinocerebellar disease, and myelitis according to the International Classification of Diseases (ICD) 8, 9, and 10. The search was made until December 2013
2. The Swedish Multiple Sclerosis Registry (SMSreg) is one of the national quality registries in the Swedish health and medical services. It started in 1996 to promote MS research and the quality of MS care (https://neuroreg.se/multipel-skleros.se). Patients with MS and possible MS according to the McDonald criteria have been prospectively and retrospectively registered and followed up at each visit [14, 17–19]. The search was made for individuals attending the neurological department in Malmö at the Skåne University Hospital
3. The Swedish cause of death registry has been computerized since 1961. We selected all cases with multiple sclerosis or inflammatory disease of the central nervous system as the underlying or contributory cause of death for individuals who died in the county of Malmö in 2001-2010.

Case retrieval from the NPR and the Swedish cause of death registry was done in September 2014 and from the SMSreg in November 2020.

(4) Cases identified by the first author at clinical work 2016-2019

### 2.4. Case Ascertainment

#### 2.4.1. Medical Chart Review

The diagnosis and year of onset were assessed by medical chart review including neuroradiological examinations such as magnetic resonance imaging (MRI), cerebrospinal fluid (CSF) analyses, and relevant laboratory tests. Cases were classified according to the 2010 McDonald's diagnostic criteria. The medical chart review was performed by the first author between October 2014 and February 2021. In order to preserve the integrity of the individuals, the National Board of Health and Welfare did not allow the authors make interviews or new examinations to confirm or refute a diagnosis of MS.

Individuals with medical records not available or incomplete were excluded. Medical records were judged incomplete when the available records were insufficient for diagnostic classification.

#### 2.4.2. The Swedish Total Population Register (TPR)

Since 1947, every individual who has resided in Sweden on a permanent basis has been recorded in the TPR kept by Statistics Sweden (http://www.scb.se). Each individual has been assigned a mandatory personal identity number. This 10-digit unique number, which includes the date of birth, the gender of the individual, and information about the time for moving or migration, makes it possible to link registries and to follow an individual over time. TPR was used to determine each individual's residence for the year of disease onset and the prevalence date.

### 2.5. Statistical Methods

For statistical calculations, SPSS 25.0 was used. The Poisson distribution was used for calculation of 95% confidence intervals (CI) for the incidence, and Wilson's score method was used for calculation of 95% confidence intervals for the prevalence. For median values, the interquartile range (IQR) was calculated.

### 2.6. Ethics

The study was approved by the local ethics committee of the Lund University, Lund (Dnr 2013/890,) and the National Board of Health and Welfare (Dnr 10576/2014). This manuscript adheres to the applicable Strengthening the Reporting of Observational studies in Epidemiology (STROBE) statement [20].

## 3. Results

**Figure 3:**
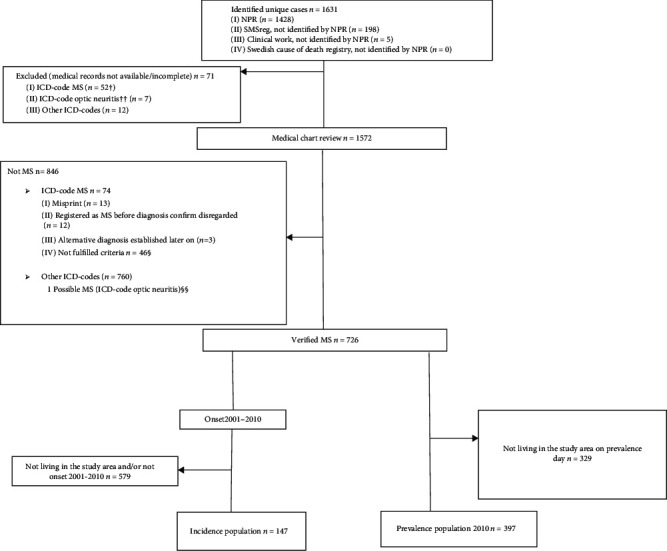
Flowchart of case retrieval and ascertainment. NPR: National patient register; MS: multiple sclerosis; SMSreg: Swedish MS registry; PPMS: primary progressive MS. ^†^Twenty-five out of 52 resident in the study area on the prevalence day, 12 out of 52 with PPMS diagnosis (all of them with onset before the incidence study period, 5 out of 12 living in the study area on prevalence day). ^††^All with onset within the incidence study period and living in the study area at that time. All living in the study area on prevalence day. ^§^Thirty out of 46 had possible MS and 2 out of 30 with onset within the incidence study period and living in the study area at that time. ^§§^Onset within the incidence study period and living in the study area at that time.

The case retrieval resulted in 1631 unique cases, of which 726 were included in the study. The vast majority (87.5%) were identified through the NPR search (Figure 3).

### 3.1. Incidence

A total of 147 incident MS cases during the period 2001-2010 were identified. The crude incidence during this period was 5.3/100,000 (95% CI, 4.5 to 6.2). The female incidence was 6.6/100,000 (5.3 to 8) and the male incidence was 3.9/100,000 (2.9 to 5) resulting in a female to male ratio of 1.8. Age-specific incidence rates are shown in Table 1. The median age at disease onset was 31 (IQR 27-38) for both males and females. There was a relapsing onset in 90.5% (*n* = 133), and the remainder had a progressive course from the start.

### 3.2. Prevalence

The onset-adjusted prevalence of MS in Malmö County on 31 December 2010 was 397 cases in a population of 298,963, giving a crude prevalence of 133/100,000 (95% CI, 120 to 146). The female prevalence was 175/100,000 (154 to 196) and the male prevalence was 89/100,000 (73 to 104) resulting in a female to male ratio of 2.1 (Table 2).

The median age at prevalence day for the prevalence cohort was 46 (IQR 35-59), 44 (IQR 33.8-55.3) for males and 47 (IQR 35-59) for females.

## 4. Discussion

There are several regional MS surveys in Sweden, but this is the first population-based epidemiological study of MS in Skåne, the most southwestern part of the country. We found a crude incidence of 5.3/100,000 (95% CI, 4.5 to 6.2), which is lower than expected according to a previous nationwide study from 2008 [7], but in line with a regional survey from Västerbotten that comprised a similar period [8]. We found an onset-adjusted prevalence of 133/100,000 (95% CI, 121 to 147), which is lower than the one in Västerbotten in northern Sweden in the same year: 215/100,000 (95% CI, 198 to 233) [8]. Thus, our results support the north-south gradient of MS prevalence in Sweden found in the nationwide survey mentioned above. However, the prevalence in our study was lower than expected according to the national figures [6].

A strength of the present study is thorough case retrieval and validation process including medical record review and strict application of diagnostic criteria. We found 46 individuals with MS diagnosis who were found to not fulfill the McDonald's criteria. A possible MS diagnosis could be considered in 30 of these. Most with relapses had had diffuse symptoms and normal or inconclusive MRI.

Among cases with medical records not available or incomplete, there were 52 individuals registered as MS according to the ICD-10 with 12 of them registered as PPMS, their clinical course was compatible with progressive disease, but they were excluded since they had not performed any MRI. Five of them were living on the study area on prevalence day. The diagnosis and differential diagnosis of primary progressive MS (PPMS) can be a challenge, with a broad spectrum of disorders to consider [21]. In addition, there were 7 individuals who had received a diagnosis of optic neuritis (ON) during the incidence period, all of them living in the study area on prevalence day. For none of these we found medical records allowing us to judge whether the McDonald criteria were fulfilled, or not.

Population-based epidemiological studies of MS in Sweden have previously been done in different counties [8–12] but not in Skåne, the most southwestern part of the country. The 2010 regional survey from Västerbotten in northern Sweden [8] covered a similar period and had, to some extent, the same methodology such as multiple sources to identify MS cases and the validation of the diagnosis by a neurologist. The median age at disease onset was somewhat lower in Malmö, and the proportion of patients with progressive course from the start was slightly higher in Västerbotten. The prevalence in Västerbotten was clearly higher than in Malmö which also supports the theory of a latitudinal gradient of MS prevalence in Sweden.

Both the incidence (2001-2008) and prevalence (2008) nationwide surveys were performed by linking register data from the NPR, the TPR, and the SMSreg but without validation of the diagnosis. Moreover, individuals with possible MS were included [6, 7]. We found a lower incidence than expected according to the nationwide figures: 5.3/100,000 respective 10.2/100,000. The national estimates were based on the registered date of diagnosis while we used onset-adjusted incidence since it is more relevant, including from an epidemiological viewpoint. In addition, we used the modified definition of onset symptoms from Poser to enable a nonarbitrary ascertainment [15]. Most epidemiological studies do not define this item at all preventing the comparability of different studies.

As regards to the nationwide prevalence, it had been estimated to be 189/100,000 with a north-south gradient ranging between 227 and 168/100,000 [6]. The figure for Skåne region was 176/100,000, but figures specific for the City of Malmö were not obtainable (Ahlgren C, personal communication). The prevalence estimation (133/100,000) in the present study is thus lower compared to the nationwide study, which may be explained by the methodological differences discussed above.

There are some limitations in our study. First, there were a few medical records incomplete or not available at all. Particularly interesting is the case of individuals with PPMS and/or optic neuritis diagnosis. One may argue that at least some of them would be likely to fulfil the McDonald's criteria if relevant tests would have been performed. Unfortunately, we were not able to make interviews or new examinations to confirm or refute the diagnosis. However, including those with PPMS and optic neuritis we are aware of being residents in the study area on prevalence day (*n* = 12) would give just a slightly higher prevalence (137/100,000), still supporting the presence of a latitudinal gradient of MS prevalence in Sweden. Second, we had no data from primary care centers. Even if most of MS care in Sweden is in the specialized health service, there may had been patients with benign disease with or without established diagnosis that have never been referred to or followed by a neurologist. Moreover, there might have been individuals with suspected or even established MS diagnosis before 1987, when the NPR register started, but no renewed contact with inpatient care after 1987 or outpatient care after 2001 and thus not possible to identify during the case retrieval. These two groups were not either included in the nationwide study.

To conclude, this is the first population-based epidemiological study in Skåne, the most southwestern part of Sweden showing a high incidence and prevalence. The incidence was lower than expected according to previous nationwide figures, probably due to methodological differences between the studies. Together with previous Swedish MS epidemiological studies, our findings support the presence of a north-south gradient of MS prevalence in Sweden.

## Figures and Tables

**Table 1:** Annual incidence 2001-2010 of multiple sclerosis in Malmö municipality per 100000 population by age and gender.

	Males		Females		Total	
Age group	Number	Rate	Number	Rate	Number	Rate
0–14	0	0	1	0.5	1	0.2
15–24	7	4.1	17	9.2	24	6.8
25–34	28	11.1	43	17.5	71	14.2
35–44	14	7.1	20	11.0	34	8.9
45–54	3	1.8	10	6.1	13	3.9
55–64	1	0.7	3	2	4	1.3
65–74	0	0	0	0	0	0
75-	0	0	0	0	0	0
Total	53	3.9	94	6.6	147	5.3
95% CI		2.9 to 5		5.3 to 8		4.5 to 6.2

CI: confidence interval.

**Table 2:** Prevalence on 31 December 2010 of multiple sclerosis in Malmö municipality per 100000 population by age and gender.

	Males		Females		Total	
Age group	Number	Rate	Number	Rate	Number	Rate
0–14	0	0	0	0	0	0
15–24	9	48	9	44	18	46
25–34	25	87	53	186	78	136
35–44	34	158	53	273	87	212
45–54	27	149	58	337	85	240
55–64	16	105	55	347	71	228
65–74	14	131	26	213	40	175
75-	5	58	13	86	18	76
Total	130	89	267	175	397	133
95% CI		73 to 104		154 to 196		120 to 146

CI: confidence interval.

## Data Availability

Anonymised data that support the findings of this study are available from the corresponding author upon reasonable request.
